# Patterns and persistence of use, effectiveness, safety, clinical inertia, and adherence related to Levothyroxine treatment with real-world evidence. An observational, longitudinal and retrospective study

**DOI:** 10.1007/s12020-025-04488-1

**Published:** 2026-02-16

**Authors:** Luis Fernando Valladales-Restrepo, Diego Andrés Londoño-Cano, Carlos Mauricio Muñoz-Velez, Lian Manuela Soto-Romero, Marlon Eleazart Tafur-Ramirez, Maria José Rojas-Varón, Jorge Enrique Machado-Alba

**Affiliations:** 1 Grupo de Investigación en Farmacoepidemiología y Farmacovigilancia, Universidad Tecnológica de Pereira-Audifarma S.A, Pereira, Pereira, Risaralda Colombia; 2 Grupo de Investigación Biomedicina, Facultad de Medicina, Fundación Universitaria Autónoma de las Américas. Institución Universitaria Visión de las Américas, Pereira, Colombia; 3 Semillero de Investigación en Farmacología Geriátrica, Grupo de Investigación Biomedicina, Facultad de Medicina, Fundación Universitaria Autónoma de las Américas. Institución Universitaria Visión de las Américas, Pereira, Colombia

**Keywords:** Levothyroxine, Hypothyroidism, Medication adherence, Inappropriate prescribing, Pharmacoepidemiology

## Abstract

**Purpose:**

Over- and undertreatment of chronic diseases are common. In this context, real-world data on the use of levothyroxine are limited. The aim was to determine the patterns of use, persistence, effectiveness, safety, clinical inertia, and adherence related to levothyroxine treatment in a group of patients with thyroid disease in Colombia.

**Materials and methods:**

This was an observational study of patients treated with levothyroxine. Clinical records were reviewed, and the minimum patient follow-up was one year. Descriptive, bivariate and multivariate analyses were performed.

**Results:**

A total of 398 patients were identified; the median age was 53.5 years, and 71.1% were women. A total of 76.9% of the patients received levothyroxine for clinical hypothyroidism, and few adverse events occurred (4.3%). 52% of the patients were not on goal at their first thyroid-stimulating hormone (TSH) checkup. Only 36.4% had a second TSH control appointment, but among them, 65.1% did not reach their goals; moreover, 31.7% demonstrated clinical inertia. The medication possession ratio was 66.8% (≥ 80%: 31.4%), and 47.7% of the patients showed persistent use at one year. The presence of adverse events was associated with a lower probability of persistent use (OR:0.16; 95%CI:0.04–0.64).

**Conclusions:**

Clinical inertia and poor disease control were common, whereas rates of adherence to and persistence with levothyroxine treatment were low. However, patients aged ≥ 40 years, those with a high educational level and chronic comorbidities, and those who habitually attended TSH control appointments were more likely to be persistent with their treatment at the one-year follow-up.

**Supplementary Information:**

The online version contains supplementary material available at 10.1007/s12020-025-04488-1.

## Introduction

Thyroid hormones are essential for the normal development and growth of many tissues and regulate the metabolism of cells and organs throughout life [1]. Hypothyroidism is a pathological condition characterized by a deficiency in thyroid hormone (including thyroxine—T4—and triiodothyronine—T3) [1, 2]. The most common cause of hypothyroidism is chronic autoimmune thyroiditis; however, other causes include radioiodine (I-131) treatment and partial or total thyroidectomy (post-surgical hypothyroidism) [1, 2]. This disease is one of the most common endocrine disorders, with a global prevalence ranging from 0.2% to 5.3% [3]. Additionally, it is estimated that its prevalence among pregnant individuals is 2.0% [3]. Thyroid hormone deficiency can affect any organ and produce heterogeneous signs and symptoms that are usually nonspecific [1, 2]. These clinical manifestations can range from mild cases with few or no symptoms (e.g., subclinical hypothyroidism) to very severe forms that can result in death (e.g., myxedematous coma) [1, 2].

Monotherapy with levothyroxine (a synthetic form of T4) is the treatment of choice for patients with hypothyroidism [1, 2, 4]. The drug improves the results of thyroid function tests and alleviates symptoms in most patients [1, 2, 4]. In addition, in pregnant women with hypothyroidism, levothyroxine reduces the risk of spontaneous abortion, premature birth, hypertensive disorders, low birth weight and impaired intellectual development in the offspring [1, 4, 5]. Moreover, levothyroxine has a good safety profile [1, 2] and is easily accessible [1, 2]. People with hypothyroidism treated with levothyroxine usually require adjustments in the doses of the drug [4]. However, over- and undertreatment in patients with levothyroxine prescriptions are common [6]. Chronic, noncommunicable disease control is dependent on factors such as persistence with and adherence to medications, the clinical inertia of the treating physician, and the occurrence of adverse drug events [6–8]. It also depends on the underlying cause of hypothyroidism. For example, in patients who have undergone thyroidectomy, achieving the optimal levothyroxine dose is more challenging, and only one-third reach euthyroidism at their first follow-up visit [9].

The Colombian Health System includes a health benefits plan that provides universal coverage to all people through two regimes, contributory and subsidized. The contributory regime is paid between people with a work contract and their employers, by independent workers with the ability to pay and by retired individuals. The subsidized scheme, in contrast, is financed by the state and covers people without the ability to pay. The health benefits plan is the same for both regimens and includes different pharmacological forms and presentations of levothyroxine, all of which are covered by the national health system (meaning that patients do not have to pay for them) [10]. Information on the use of levothyroxine in the real world is limited, and data on the persistence of its use and related clinical inertia are lacking in low- to middle-income countries such as Colombia [11, 12]. The objective of this study was to determine the patterns of use, persistence, effectiveness, safety, clinical inertia, and adherence related to treatment with levothyroxine in a group of patients with thyroid disease in Colombia.

## Materials and methods

### Study design and patients

This was an observational, longitudinal and retrospective study of patients who began Pharmacological management with levothyroxine. The patients were identified from a population-based drug dispensing database that collects information from approximately 9.3 million people affiliated with the Colombian health system. This includes patients affiliated with a private insurer, which covers approximately 3.9 million people throughout most regions of the country, and those affiliated with the contributory (85.0%) and subsidized (15.0%) regimes of the Colombian health system. The study was conducted in accordance with the strengthening reporting of observational studies in epidemiology (STROBE) guidelines

Patients aged 18 years or over, of any sex or city of residence and who began treatment with any pharmaceutical form of levothyroxine between January 1 and December 31, 2023, were included. For each subject, the date that the levothyroxine was first dispensed was considered the index date. Patients without clinical records and those without a thyroid-stimulating hormone (TSH) report obtained prior to the index date or during the follow-up period were excluded. Patients who received two different pharmaceutical forms of levothyroxine and those who were prescribed levothyroxine during the 2 years prior to the index date (that is, between January 1, 2021, and December 31, 2022) were also excluded.

**Fig. 1:**
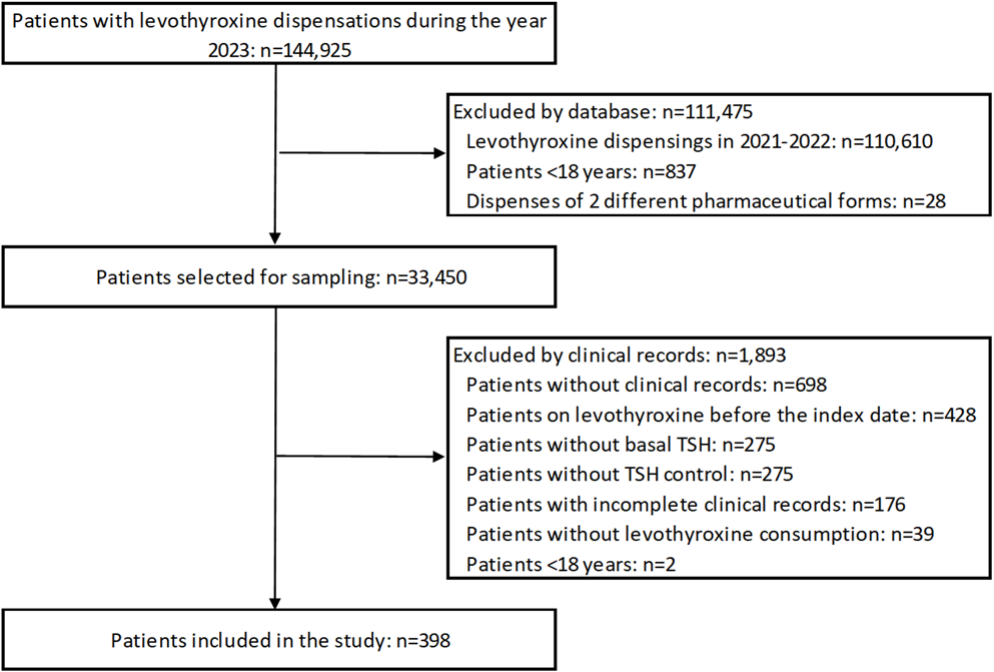
Study flow diagram

A representative sample of 398 patients was calculated from a population of 33,450 subjects via the Epi Info program (Fig. 1). The sampling was random and stratified by geographic region (proportional); the calculated sample size considered an error rate of 5%, a confidence level of 95% and an expected frequency of 50%. From the index date, follow-up was conducted for 12 months or earlier if the patient had died or had definitively discontinued levothyroxine. The electronic outpatient care records of the selected patients were manually reviewed.

### Variables

On the basis of the information obtained from the clinical records from the insurer and the drug dispensing company (Audifarma SA) [13], a database was developed that allowed collection of the following groups of variables:

1. *Sociodemographic*: Age, sex, education, occupation (with or without work activity), affiliated health system regime (contributory or subsidized) and place of origin, the last of which was categorized into the regions of Colombia according to the classification of the National Administrative Department of Statistics (DANE) of Colombia as follows: Bogotá-Cundinamarca region, Caribbean region, Central region, Pacific region and Eastern-Amazonia-Orinoquía region.
2. *Clinical*:

- Vital signs: systolic blood pressure, diastolic blood pressure and heart rate at the time of initial care and at the end of follow-up.
- Anthropometric measurements: weight, height and body mass index (BMI).
- Comorbidities: cardiovascular, endocrine, respiratory, neurological/psychiatric, rheumatological and digestive pathologies were identified, and the Charlson comorbidity index (CCI) was calculated.
- Mortality during the one-year follow-up.

3. *Clinical laboratory*: TSH level, FT4 level, hemoglobin level, hematocrit, lipid profile, glycosylated hemoglobin level, glycemia and creatinine level. The glomerular filtration rate (GFR) was calculated with the CKD-EPI 2021 equation.

4. *Pharmacological*:

- Indications for levothyroxine: clinical hypothyroidism, subclinical hypothyroidism, cretinism, TSH-dependent thyroid cancer, gestational hypothyroidism, and myxedema.
- Initial prescribing physician: general practitioner or specialist physician (e.g., internist, endocrinologist, geriatrician, family physician, gynecologist, among others).
- Initial dose of levothyroxine and dose changes reported in the clinical records during follow-up.
- Safety: adverse events recorded in the medical records (e.g., nausea, emesis, diarrhea, dizziness, palpitations, among others).
- Treatment goal: A TSH level between 0.5 and 4.5 µIU/ml; values below this range were considered to indicate uncontrolled hypothyroidism.
- Clinical inertia: defined as no change in the patient’s therapy despite an inability to reach the treatment goal.
- Adherence to levothyroxine: calculated with the medication possession ratio (MPR) formula: MPR = days that the supply of the drug was dispensed/days from the first dispensation to the end of follow-up × 100) [14]. Adherence was defined as an MPR ≥ 80.0% [14].
- Persistence: defined as the continuous use of levothyroxine during the year of follow-up and the acceptance of a grace period (gap) between dispensations < 90 days; in other words, patients who were not dispensed levothyroxine for ≥ 90 days were considered to be nonpersistent [14]. Persistence was considered the main outcome of the study.
- Comedications: after the number of medications received from the index date to the next 30 days was identified, patients were categorized as without (< 5 drugs) or with polypharmacy (≥ 5 drugs). The comedications received during the follow-up were grouped into antidiabetics, antihypertensives and diuretics, lipid-lowering agents, inhaled bronchodilators and glucocorticoids, antiplatelet agents, anticoagulants and psychotropics (antidepressants, antipsychotics, benzodiazepines and anticonvulsants).

## Results

### Sociodemographic characteristics

A total of 398 patients who met the inclusion and exclusion criteria were included (Fig. 1). A total of 71.1% of the patients were women, and the median age was 53.5 years (IQR:36.0–69.0 years). A total of 28.9% (*n* = 115) were < 40 years old, 39.4% (*n* = 157) were between 40 and 64 years old, and 31.7% (*n* = 126) were ≥ 65 years old. The highest level of education of most of the patients was secondary school (*n* = 199, 50.0%), followed by primary school (*n* = 112, 28.1%) and university (*n* = 61, 15.3%). Most of the patients were involved in some work activity (*n* = 353; 88.7%). The patients came mainly from the Central region and were primarily affiliated with the contributory scheme of the country’s health system (Table 1).

### Clinical characteristics

A total of 13.6% (*n* = 54) of the patients were pregnant. A total of 61.1% (*n* = 243) of the patients had a CCI ≥ 1 point. The most frequent comorbidities were endocrine in nature (*n* = 236, 59.3%), followed by cardiovascular (*n* = 154, 38.7%), neurological-psychiatric (*n* = 84, 21.1), digestive (*n* = 73; 18.3%), respiratory (*n* = 38; 9.5%) and rheumatological (*n* = 29; 7.3%) comorbidities. Dyslipidemia was the most prevalent comorbidity (Table 1). Only two patients had a history of thyroidectomy, and none had received I-131 therapy or antithyroid drugs in the past year. There were no statistically significant differences in systolic blood pressure (112.0 mmHg vs. 112.0 mmHg; *p* = 0.359), diastolic blood pressure (70.0 mmHg vs. 70.0 mmHg; *p* = 0.241), heart rate (77.0 bpm vs. 76.0 bpm; *p* = 0.280) or BMI (20.8 kg/m2 vs. 21.0 kg/m2; *p* = 0.715) between the beginning of follow-up and the end of follow-up. The mortality rate was 0.5% (*n* = 2).

**Table 1:** Sociodemographic, clinical and laboratory characteristics of a group of patients with thyroid disease treated with Levothyroxine in Colombia

Variables	*n* = 398	%
Sociodemographics	-	-
Female, *n* = 398	283	71.1
Age, median (IQR), *n* = 398	53.5 (36.0–69.0)	
≥ 65 years	126	31.7
Origin, *n* = 398	-	-
Central Region	170	42.7
Bogotá-Cundinamarca Region	96	24.1
Caribbean Region	51	12.8
Eastern Region	41	10.3
Pacific Region	29	7.3
Orinoquia-Amazon Region	11	2.8
Affiliation status, *n* = 398	-	-
Contributory	308	77.4
Subsidized	90	22.6
**Clinics**	-	
Vital signs (baseline), median (IQR)	-	
Systolic blood pressure (mmHg), *n* = 398	112.0 (110.0-120.0)	
Diastolic blood pressure (mmHg), *n* = 398	70.0 (66.8–78.0)	
Heart rate (bpm), *n* = 398	77.0 (72.0–80.0)	
Anthropometric measurements (baseline), median (IQR)	-	
Weight (kg), *n* = 398	67.0 (58.0-77.3)	
Body mass index (kg/m2), *n* = 398	20.8 (18.6–23.7)	
Charlson Comorbidity Index, median (IQR)	1.0 (0.0–4.0)	
Dyslipidemia	207	52.0
High blood pressure	145	36.4
Diabetes mellitus	77	19.3
Chronic gastritis	43	10.8
Chronic kidney disease	37	9.3
**Clinical laboratory results (baseline)**,** median (IQR)**	-	
Thyroid-stimulating hormone (mIU/L), *n* = 398	8.6 (6.1–12.3)	
FT4 (ng/mL), *n* = 162	0.8 (0.7–0.9)	
Hemoglobin (g/dL), *n* = 193	13.5 (12.4–14.4)	
Hematocrit (%), *n* = 193	41.0 (37.1–44.0)	
Total cholesterol (mg/dL), *n* = 240	187.5 (156.3–218.0)	
LDL cholesterol (mg/dL), *n* = 222	110.4 (82.3-135.3)	
HDL cholesterol (mg/dL), *n* = 233	46.0 (38.0–55.0)	
Triglycerides (mg/dL), *n* = 244	140.0 (101.0-196.0)	
Glycosylated hemoglobin (%), *n* = 122	5.8 (5.5–6.5)	
Blood glucose (mg/dL), *n* = 246	92.0 (85.0-101.0)	
Creatinine (mg/dL), *n* = 189	0.9 (0.8–1.1)	
GFR (mL/min/1.73 m2), *n* = 189	79.3 (65.8–95.8)	

IQR: Interquartile Range (25th − 75th Percentile); GFR: Glomerular Filtration Rate

### Pharmacological characteristics

Levothyroxine was prescribed mainly by a general practitioner and was predominantly indicated for the management of clinical hypothyroidism (Table 2). The most commonly used initial dosages were 25 mcg/day (*n* = 180, 45.2%) and 50 mcg/day (*n* = 171, 43.0%). The median initial dose in mcg/kg was 0.61 (IQR: 0.39–0.82). A total of 42.0% (*n* = 167) of the patients started with low dosages of levothyroxine. The median follow-up was 365.0 days (IQR: 237.5–365.0), and 4.3% (*n* = 17) reported some adverse events (Table 2). The median time between the index date and the date of the first TSH control checkup was 99.0 days (IQR: 64.0–168.5). 52% (*n* = 207/398) did not reach their TSH goals, and 18.6% (74/398) presented with clinical inertia. A total of 36.4% (*n* = 145) had a second TSH control checkup. The median time between the index date and the date of the second TSH control checkup was 215.0 days (IQR: 156.0–288.0). Among these patients, 64.1% (*n* = 93/145) had a TSH level outside of their goals, and 44.1% (*n* = 64/145) presented with clinical inertia (Table 2). Some patients, despite achieving a TSH level within the goal range, had their doses of levothyroxine altered (Table 2).

A total of 31.7% (*n* = 126) of patients had clinical inertia at the time of their first or second TSH control checkup (Table 2). Clinical inertia was more common in nonpregnant patients than in pregnant women (34.3% vs. 14.8%; *p* = 0.004). Almost half of the patients were persistent with their levothyroxine (Table 2). Adherence was similar between nonpregnant and pregnant women (30.2% vs. 38.9%, respectively; *p* = 0.203). The median number of medications prescribed was 4.0 (IQR: 2.0–6.0), and 42.0% (*n* = 167) of the patients had polypharmacy. The chronic comedications most commonly used during the follow-up period were lipid-lowering (*n* = 198, 49.7%), antihypertensive and diuretic (*n* = 158, 39.7%), antidiabetic (*n* = 85, 21.4%), and psychotropic medications (*n* = 75, 18.8%), antiplatelet agents (*n* = 68, 17.1%), anticoagulants (*n* = 30, 7.5%), and inhaled bronchodilators and glucocorticoids (*n* = 30, 7.5%).

**Table 2:** Pharmacological characteristics of a group of patients with thyroid disease treated with Levothyroxine in Colombia

Variables	*n* = 398	%
Initial prescribing physician	-	-
General practitioner	311	78.1
Specialist	87	21.9
Indication	-	-
Clinical hypothyroidism	306	76.9
Hypothyroidism in pregnant women	54	13.6
Subclinical hypothyroidism	38	9.5
Dose in mcg of levothyroxine (initial)	-	-
Mean ± SD	43.3 ± 21.5	
Median (IQR)	50.0 (25.0–50.0)	
Mode	25.0	
Dose in mcg/kg of levothyroxine (initial)	-	-
Mean ± SD	0.7 ± 0.3	
Median (IQR)	0.6 (0.4–0.8)	
Mode	0.7	
Control 1	398	100.0
TSH not within target range	207	52.0
Increased levothyroxine dose	101	25.4
No Levothyroxine dose change	74	18.6
Decreased levothyroxine dose	32	8.0
TSH within target range	191	48.0
No levothyroxine dose change	144	36.2
Increased levothyroxine dose	42	10.6
Decreased levothyroxine dose	5	1.3
Control 2	145	36.4
TSH not within target range	93	64.1
No levothyroxine dose change	64	44.1
Increased levothyroxine dose	23	15.9
Decreased levothyroxine dose	6	4.1
TSH at target levels	52	35.9
No levothyroxine dose modification	49	33.8
Increased levothyroxine dose	2	1.4
Reduced levothyroxine dose	1	0.7
Adverse events	-	-
Dizziness	5	1.3
Tachycardia	4	1.0
Palpitations	3	0.8
Fatigue	2	0.5
Emotional lability	2	0.5
Nausea	2	0.5
Alopecia	1	0.3
Diarrhea	1	0.3
Insomnia	1	0.3
Weight loss	1	0.3
Clinical inertia	126	31.7
Adherence	-	-
Mean (%) ± SD	68.4 ± 21.2	
Median (%) (IQR)	66.8 (53.1–83.3)	
MPR = 80%	125	31.4
Persistence at 1 year	190	47.7

SD: Standard deviation; IQR: Interquartile range (25th percentile − 75th percentile); TSH: Thyroid-stimulating hormone; MPR: Medication Possession Ratio

### Multivariate analysis

After adjustment for sociodemographic, clinical and pharmacological variables, the exploratory binary logistic regression analysis revealed that age ≥ 40 years, a high educational level, a CCI ≥ 1 point, clinical hypothyroidism and a second TSH control checkup during the follow-up period were associated with an increased probability of persistence with levothyroxine at the start of the year. On the other hand, the development of adverse events was associated with a reduction in this probability (Hosmer–Lemeshow test, *p* = 0.844) (Table 3).

**Table 3:** Logistic regression on variables related to the persistence of Levothyroxine one year after initiation, in a group of patients with thyroid disease, Colombia

Variables	*p*	aOR	95%CI	
			Lower	Upper
Female (yes/no)	0.140	0.676	0.402	1.137
Age ≥ 40 years (yes/no)	0.044	2.215	1.021	4.805
Place of origin: Bogota-Cundinamarca (yes/no)	0.816	1.067	0.617	1.845
University education (yes/no)	0.004	2.932	1.412	6.089
CCI ≥ 1 point (yes/no)	0.005	2.447	1.306	4.586
Initial prescription of levothyroxine by a general practitioner (yes/no)	0.402	1.294	0.708	2.366
Diagnosis of clinical hypothyroidism (yes/no)	< 0.001	7.127	3.323	15.284
Initial dose of levothyroxine (continuous)	0.354	0.995	0.985	1.006
First TSH monitoring at target levels (yes/no)	0.072	1.614	0.958	2.719
Second TSH monitoring (yes/no)	0.003	2.217	1.320	3.722
Clinical inertia (yes/no)	0.961	1.014	0.581	1.769
Adverse event (yes/no)	0.010	0.162	0.040	0.646

aOR: Adjusted Odds Ratio; CI: Confidence Interval; ICC: Charlson Comorbidity Index; TSH: Thyroid-stimulating hormone

## Discussion

This study revealed the way in which levothyroxine is used in a low- to middle-income country. Levothyroxine is used primarily in the management of clinical hypothyroidism. The starting dose of levothyroxine was low in many cases, and thyroid function tests indicated that most patients were not on target. Clinical inertia was common, but adverse events were uncommon. Adherence and persistence were not optimal in many of the patients. Knowledge of the use of drugs allows the development of standardized interventions with the aim of allowing prescribing physicians the ability to improve patient quality of care and thus achieve adequate clinical disease control [15]. This is even more relevant given reports by the World Health Organization (WHO) that the inappropriate use of drugs is a major and growing problem worldwide [16].

The indications for which levothyroxine was prescribed are in accordance with clinical practice guidelines and the country’s drug regulatory agency (National Institute for Food and Drug Surveillance) [17–19]. However, thyroid function tests indicated that most patients were not within adequate control ranges during follow-up. This problem has also been described in other studies [20, 21]. In Turkey, Karataş et al. reported that only 32.1% of patients had control of their TSH levels in the euthyroid range [20], whereas in the USA, Somwaru et al. reported suitable TSH level control in 43.0% of patients [21]. Poor control of hypothyroidism may be the result of different influencing factors, including the low doses with which levothyroxine is started, the clinical inertia of the treating physician and poor adherence to the drug [6–8].

The initial dosages of levothyroxine were 25 or 50 mcg per day for the vast majority of patients, indicating that many patients received low doses, which is not in line with the recommendations of the country [17]. The Colombian consensus recommends adjusting the dose according to the baseline TSH values; in this way, the dosages can range from 25 mcg to 100 mcg daily. In addition, patients with cardiovascular disease and those aged ≥ 60 years should start with 12.5 to 25 mcg per day [17]. Likewise, it was found that some patients, despite having a TSH level outside of their goals, did not experience dose adjustments. This condition is known as clinical inertia and is a frequent phenomenon that occurs during the management of chronic noncommunicable diseases [7, 22]. However, little information is available on clinical inertia in patients with hypothyroidism [7, 22]. A study of patients with thyroid cancer receiving suppressive treatment with levothyroxine revealed that clinical inertia was present in the majority of patients [23]. At the beginning of the follow-up period, a TSH level in the target range was observed in only 8.8% of the patients, while by 18 months, this percentage had increased to only 19.6% [23].

Adherence was adequate in only one-third of the patients who started levothyroxine, a finding that has also been described in other countries [24–27]. These investigations employed the Morisky–Green test instead of the medication possession ratio to quantify adherence [11, 24–26, 28]. However, the findings of this report are similar to those from studies from Pakistan (32.2%) [24], Oman (26.0%) [25] and Saudi Arabia (10.0%) [26] but contrast with the results of a study previously carried out in Colombia and those from a study in Italy, where the majority of patients were adherent to the treatment (85.8% and 87.0%, respectively) [11, 28]. In the USA, Hepp et al., used the proportion of days covered (PDC) and reported that 48.1% of the patients were adherent to the drug (PDC ≥ 80%) [27]. Adherence problems are multifactorial and depend on patient (e.g., own beliefs, comorbidities), health-system (e.g., unsatisfactory previous encounters, difficulties in meeting control appointments) and social environment factors (e.g., economic problems or access to health centers) [8].

Similarly, almost half of the patients persisted with their levothyroxine treatment. This finding is similar to that reported in the USA by Wang et al., who reported that 51.5% of patients persisted with treatment one year after initiation [29]. Older patients with a higher educational level were more likely to be persistent with treatment, which is consistent with findings in the literature [26, 30]. This may be because these patients are more aware of the importance of continuing medical management [30, 31]. Furthermore, those with a CCI ≥ 1 point were more persistent than those with a lower CCI. Patients with a greater burden of disease have a greater perception of risk, a greater need for treatment and more interactions with the health system [30–32]. Patients with a diagnosis of clinical hypothyroidism were more likely to persist with levothyroxine treatment. Not all patients with subclinical hypothyroidism are indicated for pharmacological treatment, so levothyroxine use could be suspended according to the criteria of the treating physician and scientific evidence [17, 19]. Similarly, many women who require levothyroxine during pregnancy stop receiving the drug because they are euthyroid during puerperium [5]. Finally, patients who had more control appointments involving laboratory measurements of TSH levels were more persistent with levothyroxine treatment, which is consistent with the findings of other studies [24, 26]. Laboratory-based TSH control ensures that the patient is in frequent contact with the health system and that they can tangibly observe improvements in or worsening of their pathology [31, 32]. Finally, patients who experienced adverse events were less adherent to medication management, corroborating a phenomenon that has been widely documented in the literature [31, 32]. The occurrence of adverse events leads the patient to interrupt or suspend use of the drug [32]. However, the safety profile of levothyroxine is favorable [1, 2].

Some limitations should be recognized when the results of this study are interpreted. First, for some laboratory variables, information, especially serum FT4 levels, was not available for all patients. Antiperoxidase antibody (TPOAb) and antithyroglobulin antibody (TgAb) levels are very rarely reported in this group of patients. Second, the diagnoses of the patients were based on what the treating physician recorded in medical history. Third, most patients did not have a second TSH control checkup during the follow-up period. Fourth, it is unknown whether the patients consumed all the medications and at the quantities provided by the pharmaceutical manager. Fifth, it is unknown whether the patients bought their levothyroxine with their own money. This information is not recorded in the clinical records or in the dispensing database and may influence the adherence/persistence results. Sixth, adverse events were very rarely reported in the clinical records, and therefore, establishing causal relationships with levothyroxine was not possible. Seventh, the findings cannot be extrapolated to patients with hypothyroidism secondary to thyroidectomy, given the very small number of such cases included in this study. However, the study involved a random and important sample of patients with hypothyroidism who were affiliated with the contributory or subsidized regimes of the health system and were located throughout different geographical regions of the country.

### Ethical statement

The protocol was endorsed by the Research Ethics Committee of the Technological University of Pereira in the category of “risk-free research” (approval code: 27-130323). The principles of confidentiality of information established by the Declaration of Helsinki were respected. The study was endorsed by the insurer who was responsible for custody of the patients’ information. Studies carried out with data from clinical registries do not require informed patient consent according to the norms of Colombia.

### Statistical analysis

The data were analyzed with the statistical program SPSS Statistics, version 26.0 for Windows (IBM, USA). Descriptive analyses included the determination of frequencies and proportions for qualitative variables and measures of central tendency (means, medians, mode) and dispersion (standard deviation (SD) and interquartile range (IQR)) for quantitative variables. No imputation of missing data was performed. Quantitative variables were compared with the Mann‒Whitney U test, and categorical variables were compared with the X^2^ test. Comparisons were made as follows: (1) systolic blood pressure, diastolic blood pressure, heart rate, and BMI between the beginning vs. the end of the follow-up; and (2) clinical inertia, adherence and persistence between nonpregnant and pregnant patients.

An exploratory multivariate analysis was performed via binary logistic regression. The dependent variable was persistence with levothyroxine one year after initiation (yes/no). The candidate independent variables (covariates) for the logistic regression were those that showed a statistically significant association with the dependent variable in the bivariate analyses as well as those with sufficient reported plausibility (e.g., age, sex, comorbidities). The forward selection method was used to select the variables. Adjusted odds ratios (aORs) are presented with 95% confidence intervals. The level of statistical significance was established at *p* < 0.05. The Hosmer–Lemeshow test was performed to determine the goodness of fit.

## Conclusion

On the basis of the findings of this study, we conclude that the indications for levothyroxine, but not the dosages prescribed when starting on the drug, are in line with the recommendations of the clinical practice guidelines. Clinical inertia and poor disease control were common, whereas the rates of adherence to and persistence with levothyroxine were low. However, patients aged ≥ 40 years, those with a high educational level, chronic comorbidities and a diagnosis of clinical hypothyroidism, and those who habitually attended TSH control appointments were more likely to be persistent with treatment at the one-year follow-up. Continuing education programs should be developed to improve the quality of levothyroxine prescriptions. Future pharmacoepidemiological studies should focus on patients with hypothyroidism secondary to thyroidectomy, particularly in populations from low- and middle-income countries.

## Supplementary Information

Below is the link to the electronic supplementary material.

Supplementary Material 1

## Data Availability

protocols.io DOI: dx.doi.org/10.17504/protocols.io.bp2l6z39dgqe/v1 (Private link for reviewers: https://www.protocols.io/private/171AE91494DD11F096700A58A9FEAC02 to be removed before publication.)
