# Supplementary figures and images for: Patterns and persistence of use, effectiveness, safety, clinical inertia, and adherence related to Levothyroxine treatment with real-world evidence. An observational, longitudinal and retrospective study
Source: Endocrine. 2026 Feb 16;91(1):84. doi: 10.1007/s12020-025-04488-1 (PMC12909374; doi:10.1007/s12020-025-04488-1)

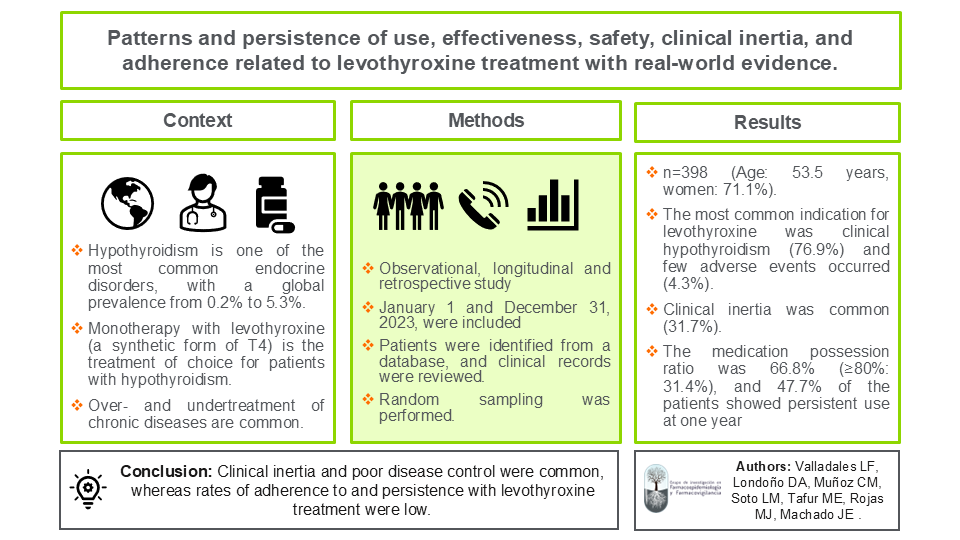

Supplement: Supplementary file 1 — Supplementary Material 1 [file 12020_2025_4488_MOESM1_ESM.tif]
